# cleAR: an interoperable architecture for multi-user AR-based school curricula

**DOI:** 10.1007/s10055-023-00764-5

**Published:** 2023-02-27

**Authors:** Stefano Masneri, Ana Domínguez, Miguel Sanz, Mikel Zorrilla, Mikel Larrañaga, Ana Arruarte

**Affiliations:** 1 Computer Languages and Systems Department, University of the Basque Country UPV/EHU, San Sebastian, Spain; 2 Fundación Vicomtech, Basque Research and Technology Alliance (BRTA), San Sebastian, Spain

**Keywords:** Augmented reality, Multi-user interactions, Collaborative learning, Visual learning analytics, Artificial intelligence

## Abstract

Although there are some experiences that demonstrate the validity of the use of augmented reality in schools to help students understand and retain complex concepts, augmented reality has not been widely adopted in the education sector yet. This is in part because it is hard to use augmented reality applications in collaborative learning scenarios and to integrate them in the existing school curricula. In this work, we present an interoperable architecture that simplifies the creation of augmented reality applications, enables multi-user student collaboration and provides advanced mechanisms for data analysis and visualization. A review of the literature together with a survey answered by 47 primary and secondary school teachers allowed us to identify the design objectives of cleAR, an architecture for augmented reality-based collaborative educational applications. cleAR has been validated through the development of three proofs of concept. cleAR provides a more mature technological ecosystem that will foster the emergence of augmented reality applications for education and their inclusion in existing school programs.

## Introduction

The usage of technology in the last decade has transformed the education sector, empowering teachers and students by creating more engaging environments, facilitating collaboration and enabling different learning styles. During the COVID-19 pandemic and its associated lockdowns, most teaching activities have moved online and all stakeholders relied on different technologies to keep school activities ongoing (Azorín 2020).

In recent years, augmented reality (AR) technology is being used more and more, thanks to an ever-increasing number of devices supporting it, as well as a more mature software ecosystem that allows developers to speedup the creation of AR-based applications. With their ability to attach virtual content to any physical surface, either through the usage of markers or by using plane detection or geographical information, AR applications have found a valuable role in training and education. Several companies offer educational AR apps and many scientific publications have shown that AR can enhance and improve the learning experience (Akçayır and Akçayır 2017).

Unfortunately though, AR has not yet seen widespread usage in education, and most of the experiences carried out so far are based on small experiments. This is due to several causes (presented in Sect. 3.2) which can be summarized with the difficulty for developers and educators to create content that can be used by every student and that integrates well with the existing school curricula. One of the main issues, for example, is that the majority of AR applications available for education provide single-user experiences and they are more apt to be consumed at home rather than at school. Cooperative learning has long been used as an educational approach to improve students’ learning and performance (Johnson and Johnson 2019; Kuh et al. 2011), but since the majority of existing AR applications are not multi-user, they do not allow students to cooperate. Another issue limiting the adoption of AR in schools is the integration of AR applications within existing school programs. Existing applications cannot be easily adapted to specific school curricula, and the data generated inside the apps (e.g., test results or lesson progress) is not automatically added to a learning management system (LMS), thus creating additional workload for teachers.

To solve these problems, we present cleAR (collaborative learning environment for augmented reality), an interoperable architecture that enables the creation of multi-user AR applications while providing advanced mechanisms for data analysis and visualization. The main contributions of this paper are the following:

- Definition of multi-user AR requirements and their translation into design objectives. We identified the requirements which an AR-based educational application should satisfy. We contacted teachers from the Basque association of primary and secondary schools (Ikastolen Elkartea) and asked them to complete an online survey. The answers and the feedback provided led us to the definition of the requirements and the design objectives.
- An interoperable architecture for multi-user AR-based apps. We defined cleAR, an architecture that allows educators and developers to design multi-user and collaborative learning experiences. Multi-users interaction allows sharing the same AR experience across users as well as transmission of information of any kind (textual, audio, video or 3D). This information could be, for example, interactions of a student in the augmented space, data gathered by the sensors in the devices, information provided by the professor from his laptop or a live video grabbed by a user with their mobile phone. cleAR also includes libraries for data analysis and data modeling. As it has been designed with ease of use as one of the core design objectives, the architecture offers the user several web-based tools for data visualization, reporting and information sharing. As the availability of hardware and software is extremely heterogeneous across different schools, the architecture supports several platforms, both web and native.
- Validation of the design objectives within the proposed architecture. We developed three proof-of-concept (PoC) applications to validate the architecture and to verify that it fulfills all the design objectives we identified. Such PoCs are released as open-source software^1^ and can be used and combined by developers to create their own AR applications. These PoCs do not include all functionalities required by full-fledged applications: for example, privacy considerations such as compliance to GDPR (or other national or local regulations), user handling or access right management are not included in the PoCs, as such functionalities are application dependent and can vary greatly depending on the scope of the app.

The rest of this paper is organized as follows: Sect. 2 presents the related work. Section 3 describes the requirements of a multi-user AR solution and how they are translated into design objectives, while Sect. 4 introduces the proposed architecture, called cleAR. Section 5 presents the PoCs implemented to validate cleAR and, finally, Sect. 6 summarizes the paper and suggests future research lines.

## Related work

In the last few years, a massive amount of publications presented implementations of AR applications for education. Several systematic literature reviews describe usage of AR in different settings, such as science, technology, engineering and mathematics (STEM) subjects (Ibáñez and Delgado-Kloos 2018), game-based learning (Pellas et al. 2019; Dinis et al. 2017), or about the status and tendencies of AR in education in general (Akçayır and Akçayır 2017; Garzón et al. 2019; Chen et al. 2017; Masneri et al. 2022). The review of Phon et al. (2014) describes ten collaborative AR applications used in education, but only four of them include collaborative features in the AR experience, while the others only use the collaborative approach as a learning strategy, which is not related to the application. Other works focus their attention on specific applications of AR, such as education for industry 4.0 (Martin et al. 2018) or medical education (Kuehn 2018).

As the support of AR technology in modern browsers is relatively recent, there is a scarcity of publications presenting web-based AR applications. The work of Abriata (2020) describes a web application enabling the creation of AR experiences for molecular visualization, while (Coma-Tatay et al. 2019) presents a solution for the creation and visualization of generic AR applications in browsers which uses the open-source software framework FIWARE.

Even though most educational AR applications described in the literature are intended to be single-user, researchers have been investigating collaborative AR experiences since the seminal publication of Billinghurst et al. (2002). In (López-Faican and Jaen 2020), the authors present a markerless AR application for improving socialization and communication skills of primary school children, and note that the collaborative game version of the app has a greater impact on emotional affection and social interaction. Oh et al. (2017) describe a collaborative AR app where the user, through the use of smart glasses, can study properties of light such as reflection and refraction. Each user acts as a light source and sees what happens when light hits a wall or passes through different materials. At the same time, two or more users can generate multiple light rays and see how they interact with each other.

Besides AR, the revolution of information and communication technologies (ICT) has affected education in many other ways, providing means to enhance both the teaching and learning processes. Nowadays, technology enhanced learning (TEL), such as intelligent tutoring systems (ITSs), adaptive hypermedia systems (AHSs), and learning management systems (LMSs), are being widely used in many schools and becoming essential for education. An intelligent tutoring systems (ITSs) is a system which aims to replicate with digital tools the effectiveness of human tutoring. Even though the first ITS was created over 50 years ago (Carbonell 1970), recent advances in artificial intelligence (AI) translated into the development of newer systems for both education and professional settings (Mousavinasab et al. 2021) that have an effectiveness comparable to that of human tutoring (VanLehn 2011). ITSs are typically composed of four components: a domain model, a student model, a tutoring model and a user interface (Nkambou et al. 2010). AI tools can be applied to each model but are especially relevant for student models, as they represent the student’s current state of knowledge and are used to provide optimal teaching interventions (Sedlmeier 2001). Student models can then be characterized by what kind of information they model and how this information is stored and used (Chrysafiadi and Virvou 2013). There are only a few studies examining the combination of ITSs and AR: in (Westerfield et al. 2015) the authors present an AR application for motherboard assembly, where the usage of an ITS allows personalized training, while the work of Schez-Sobrino et al. (2020) uses AR to create 3D graphical representations of computer programs, helping students learn new programming concepts.

Visual learning analytics (VLA) is the research area at the intersection of visual analytics and learning analytics (Therón 2020). A recent survey of studies applying VLA to educational settings shows that so far there are very few examples of bringing VLA tools into the classroom and they generally use only very simple visualizations and do not consider background student information as data source (Vieira et al. 2018). Another review study (Bodily el al. 2018) analyzes similarities and differences between open learner models (OLMs) and Learning Analytics Dashboards (LADs) and concludes that there is a strong overlap in the two fields, and that applying the lessons learned in OLM research can drive the next generation of learning analytics tools.

Despite the huge amount of literature describing AR applications for education, we were not able to find works describing systems that use AR technology, provide multi-user and collaborative functionalities and make use of visual learning analytics tools and/or intelligent tutoring systems.

## Requirements and design objectives

To better understand the requirements of an architecture that enables educators to easily incorporate collaborative AR applications in their curricula, we prepared a questionnaire for teachers of primary and secondary education level where we asked a set of questions related to the usage of technology, and AR in particular, in their schools. The survey followed the methodology recommended in Lazar et al. (2017, Chapter 5), but it was later revised following the recommendations of the teachers who revised the document, who suggested to reduce the amount of open-ended questions and to prepare a survey which would not take more than 20 min to complete. Based on the answers to the survey, we extracted a set of requirements that in turn defined the design objective of the proposed architecture.

### Teacher survey

The teacher survey^2^ we prepared is composed of 45 questions, split across three sections. Nineteen of the questions were open-ended and in many cases optional, while the remaining questions are multiple choice. The respondents did not have to answer all the questions, since the survey presented different branching paths based on the answers provided. For example, there is a set of questions asking about the teachers experience with AR applications, which are presented only to the respondents who answered positively in the question about the previous usage of AR in the classroom.

The first section of the survey contains 16 questions about the teachers (which subjects they are teaching, their years of experience, whether they teach in primary or secondary schools, etc.) and about the generic usage of technology in the classroom (how many laptops, tablets or smartphones are available at school, what applications they use besides office-related or videoconferencing solutions) and what advantages are provided by such technologies.

The second section contains 14 questions specific to the usage of AR applications at school. The section starts with a brief description of what AR is, as well as a few examples of how AR applications can be used in schools. These paragraphs were introduced so that even professors not familiar with the technology would be able to answer the questions related to AR. If the teachers had already used AR, the survey asked how often they used it, what they need to use it, on which devices and whether they were satisfied with the experience. If the teachers did not have any previous experience using AR at school, the survey asked them whether they thought that AR could be a valuable tool to facilitate learning. Furthermore, the questionnaire asked the teachers what changes would be required in order to improve the experience of using AR and whether they think that an AR-based application would improve the students’ learning experience.

The final section includes 15 questions about the technological tools the teachers would like to use in their daily activities and when and where they would like to use them. Some questions focused specifically on AR, its advantages and disadvantages, what the teachers consider as the most interesting functionalities of AR apps, what kind of content they would show in the app and their willingness to create such applications, if they were given adequate authoring tools. The final questions were about the usage of AR in a collaborative learning environment and about the inclusion of artificial intelligence as a support tool for analyzing student data and creating automatic reports.

### Survey results

The survey was answered by 47 teachers belonging to the Basque association of primary and secondary schools. In this subsection, we will briefly summarize the results of the survey, from which we then extracted the architecture requirements. The collection of anonymized answers, together with an exploratory data analysis, is available online.^3^

With respect to demographic information, the majority of the respondents (53%) teaches STEM subjects in secondary schools (82%), in classes with 25 students on average. 43% of the teachers who answered the survey are “facilitators”, that is teachers in charge of helping colleagues to use technical tools in the classroom, and they are usually the most tech-savvy among the school personnel.

Schools are usually provided with many computers: although there is a lot of variance across the answers provided, on average each school has 68 desktop PCs available as well as 398 laptops. Tablets are seldom used (42% of the teachers said they have no tablets in school, but others mentioned an availability of up to 75 tablets). Every teacher mentions they have mobile devices available, but in most cases only their personal device. Teachers also mentioned that most of the students have a personal device, depending on their age (which was not asked in the survey). Apart from this, the teachers mentioned that they often use devices such as Chromebooks or Ultrabooks, digital blackboards, projectors or Apple TVs. From this, a solution that will work on top of different platforms and devices is extracted as a requirement (Requirement 1—R1).

Regarding software, 77% of the respondents use software tools (besides Office applications) every school day. The most used applications are Moodle, the Google suite (Classroom, Drive, YouTube, Earth, Maps), SketchUp, Scratch, GeoGebra, Prezi and Lucidpress. The teachers use these applications because they help motivate the students and achieve teaching objectives. The most appreciated aspects of these applications are as follows (in descending order):

- They provide services which are accessible from different devices (86% of the respondents). (R1).
- Foster the interaction between the teacher and the students (86%) (R2)
- Foster the interaction between multiple students (75%) (R3)
- Provide customization options (language, content, etc.) (69%) (R4)
- Gather data on how the application was used (53%) (R5)

Apart from these aspects, the teachers underlined as relevant aspects the possibility to update and maintain the tools (both hardware and software), providing the tools for free and using hardware with enough storage capacity (R6).

Regarding AR, the vast majority of the respondents (88%) never used it at school, and only about 3% used it more than once and very seldom. The question specifically mentions the usage of AR in school, and the high percentage does not reflect the teachers’ familiarity with the technology or their usage of AR in other contexts. Among those who used it, 75% of the teachers think that AR improved the students’ learning process. Despite the low usage of AR technology, 78% of the teachers think that AR can be a very useful tool. When asked what could help in increasing AR adoption in schools, about 75% of the teachers emphasized they would need to know the existing AR ecosystem better, what apps are available and their capabilities. Half of the teachers said that they would need more time and to have better hardware and software available for the students (R7). Other answers mentioned the necessity of technical support, better localization of the content and the ability to support multi-user interactions (R8), as the students get bored fairly soon doing individual activities. In general, teachers are dissatisfied with AR for education, rating it 2.2 on a Likert scale (where 1 means “very low satisfaction” and 5 means “very high”). The reasons for this are because most of the AR apps are very limited in terms of interactivity and user experience, and there is nearly no content in the Basque language. Despite this, 70% of the respondents think that AR can be of added value in learning, and the remaining 30% think that it may be. The main advantages of AR, as identified by the teachers, are as follows:

- Improvement of the students’ motivation (82%)
- Better assimilation of concepts (78%)
- Better way to transfer knowledge (69%)
- Improvement of spatial orientation (58%)
- Improvement of interactivity (49%)

On the other hand, the teachers identified several key limitations of AR, as it requires an effort to learn how to use the technology and the school curricula must be adapted to include it. Furthermore, teachers often lack the time to get familiar with the technology and the experience is often not engaging enough, either because the devices used are too old or because the apps lack content. Ideally, the teachers would like to use AR applications that:

- Enable collaboration between multiple students as well as with the teacher
- Are highly interactive and with plenty of quality content
- Can work with a broad range of devices
- Can be used both in the classroom and remotely
- Collect data about how the students used the app

The teachers also expressed interest in having the possibility to create their own AR content, if they were provided an authoring tool and training on how to use it. More than half of the respondents (53%) expressed their interest and that they would like to create simulations, quiz activities, immersive videos and 3D visualizations. Some of them mentioned that they routinely use tools such as Kahoot for creating interactive web quizzes and they could use something similar for the creation of AR experiences.

Finally, regarding the usage of AI in education, 40 teachers identified the following use cases:

- Analysis of usage data and usage patterns (63%) (R9)
- Automatic analysis of the difficulty of the questions in a test-type activity (60%) (R10)
- Identification of students with difficulties at an early stage (58%) (R11)

**Table 1:** Summary of the requirements identified from the results of the teacher survey

Code	Requirement
R1	Apps should work on different platforms and devices
R2	Foster teacher-student interactivity
R3	Foster student-student interactivity
R4	Enable several customization options
R5	Collect data on how the applications are used
R6	Enable easy development as well as installing and updating
R7	Apps should work smoothly even on older hardware
R8	Apps should allow collaborative work
R9	Provide tools for analysis of data and usage patterns
R10	Enable automatic analysis test difficulty
R11	Detect students with learning difficulties

Table 1 recapitulates all the architecture requirements identified after analyzing the answers to the survey.

### Design objectives

**Fig. 1:**
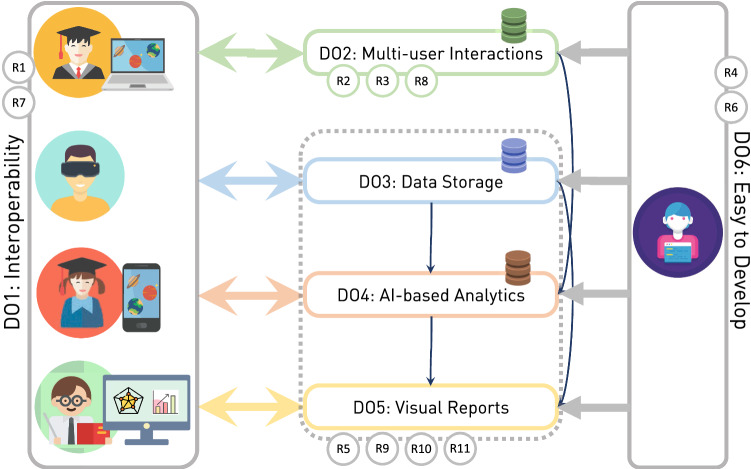
A diagram representation of the architecture design objectives

Based on the teachers’ answers collected in the survey and the identified requirements, we derived six design objectives (summarized in Fig. 1) that guide the definition of an architecture for AR-based applications which can be used in schools:

- The architecture must be *interoperable*—Design objective 1 (DO1)—both at the hardware level, as it should run on different devices such as head-mounted displays (HMDs), tablets, laptops or smartphones, with reasonable support for older models, and at the software level, as ideally the architecture should provide application programming interfaces (APIs) for development on native platforms (Android and iOS), for cross-platform engines like Unity and support Web standards for extended reality (XR) experiences (Goregaokar et al. 2022) and real-time communication (Holmberg et al. 2015). An interoperable architecture has two advantages: it eases the dependence on specific hardware, thus reaching a wider user base, and it allows the development of hybrid solutions where users can connect either using an app or their browser.
- The architecture should support *multi-user interactions* (DO2), both in-person and remote. Collaboration is a key requirement identified by teachers to increase the engagement of the students and keep them interested, so the architecture should enable user communication (via voice or chat) and also provide a way to exchange any kind of data in real time, for example the interactions of the users with the application, the position of the AR camera, the answers to the questions in the app, etc.
- The architecture should enable *long-term storage* (DO3) of the data collected, to guarantee that the teachers can track the progress of the students over time and to let them create *data visualizations* or automatic reports (DO4). The data will enable the implementation of AI techniques (DO5). The architecture gathers information about how the students are using the applications (both the interactions with the software as well as with other users) and store it in the appropriate format. The architecture should be agnostic to the AI models built on top of it (depending on the application, teachers may be interested in using recommender systems, anomaly detection systems or clustering algorithms, for example) but it should provide support for training a model from scratch, fine-tuning a previously trained model or using an existing model for inference tasks.
- Finally, it should be *easy to develop* content using the specified architecture (DO6). Simplifying the development process would hopefully encourage developers to create content based on the architecture, thus solving the problem of the lack of quality content identified by the teachers. The architecture should also be *easy to use*, simplifying the deployment and maintaining of the apps: in the ideal case the teacher using the AR application would be able to install and update the software for himself and the students without the need of external help. It should be noted though that this design objective is not geared toward the creation of an authoring tool but rather on the definition of a set of libraries and APIs that allow software developers to speedup the app creation process. There is a lack of teacher-oriented authoring tools: most of them have been developed for computing literate users or knowledge engineers and, therefore, they become too complicated for teachers, who may give up the development of their own applications. Brusilovsky et al. (2003) claim that teachers should focus on Domain Module authoring while the development of the core of technology support learning systems should be carried out by expert developers.

## Architecture definition

In this section, we describe cleAR, the architecture that fulfills all the design objectives described in Sect. 3. It is a client–server architecture composed of several modules, each of which is in charge of a specific task. Two design objectives, namely interoperability and ease of development, are not satisfied by specific modules but are rather fulfilled thanks to how the architecture has been designed. For interoperability, the definition of a web architecture and the development of multiplatform libraries ensures that developers can create applications working on different hardware (PCs, HMDs, tablets or mobile phones) and across multiple software tools, as the architecture has been tested on multiple browsers, desktop applications as well as native iOS and Android apps. While cleAR does not provide authoring tools that would allow AR applications to be created without requiring coding experience, extreme care was taken in developing a set of libraries and APIs that enables developers to easily create interoperable multi-user AR applications, as we will show in Sect. 5.

**Fig. 2:**
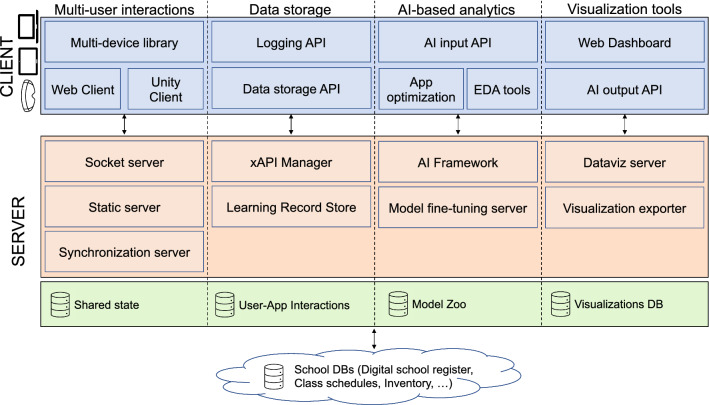
A visual description of the proposed architecture

In the following subsections, we will describe in detail the four main blocks of the proposed architecture, as summarized in Figure  2. While part of the architecture has been developed from scratch, some components rely on existing libraries to provide the required functionalities. All the components have been integrated in a cohesive system that can be used to create collaborative AR applications.

### Collaborative real-time multi-user library

This is the module enabling real-time communication between multiple users. The module relies on a server for message passing between different clients and its objective is to abstract the application code from all the nuances and difficulties of network communication. While the definition of the module is platform agnostic, we recommend the usage of WebSocket (Fette and Melnikov 2011) protocol to share data across the internet with low-latency, since several frameworks support it and thus it can be integrated in any software libraries developed, for example, with Unity or any Web framework.

On the client side, the module defines a set of library calls enabling users to connect to a shared session, through which they can exchange messages and share data of any kind such as application variables, json files or images. On top of that, the module defines different clients enabling its usage on different platforms.

On the server-side, the module introduces synchronization mechanisms that allow clients to play multimedia tracks at the same time, enabling a shared experience, and defines a static server that keeps track of the shared state (a database defining the current state of the variables shared across users) and which is responsible for notifying any state change. Finally, the library includes a socket server which is responsible for establishing the connection with the clients and manages low-level communications.

The library can also be used to share multimedia content, for example through the WebRTC protocol (Holmberg et al. 2015). In this case, the module relies on another server to handle signalling and relaying data between the server and the application. The architecture is ready to work with existing solutions such as Janus or MediaSoup, which provide all the required functionalities and can be easily integrated.

The real-time multi-user library has been designed to be simple, with the aim of reducing the latency and the time required for processing data as much as possible. Apart from the aforementioned WebSocket server and the optional WebRTC gateway, it includes a timing server to handle synchronization of the content, and a Rest API for manipulating data persistence. Connection to the server is organized via dedicated rooms, so that every user connecting to the server URI at a specific room will share the same AR experience. Rooms can be organized hierarchically and the application developers can specify the maximum amount of users per room.

### Logging and data storage

Besides sharing data and messages in real time, users may be interested in permanently storing other kinds of data. It can be data related to the user progress in specific tasks, like the answers to a test or the completion of a chapter, or other data linked to the interactions of the user within the application, for example the number of clicks, selections in a menu, or the interactions with 3D content in the augmented space.

In this case, cleAR allows storage of data that can be serialized and stored in a database or on a local disk, and it provides means for easily querying and filtering the data when needed, either directly in the application or through a script. The architecture provides an API to store and access the data through a Learning Record Store (LRS), thus simplifying its integration into LMSs already in use at school. By enabling storage of the data on an external database, applications developed with this architecture can then be integrated into the school curricula, as they can fetch data from the LMS (for example, a set of questions and answers for a test) as well as writing new data to it (e.g., the results of the in-app quizzes).

Finally, logging and storing usage data and activity can enable user monitoring practices. In the case of AR experiences, the teachers would be able to know how much time each student spends on different modules of an application, giving him or her insights on which concepts are harder to grasp. Furthermore, the teacher would be able to check if any of the students is falling behind, as the application can raise automatic flags if the student has not accessed an app in a while, or is performing consistently bad on the assessment questions. The amount of data that is stored is fully customizable at the application level, and there are options for anonymization, adding user profiles (e.g., admin, teacher and student) and for changing the frequency of data collection.

### AI-based analytics

Given the amount of data that are made available by the module described in Sect. 4.2, AI techniques, especially machine learning (ML) algorithms, can be used to build learner and group models that can improve the learning processes. The models trained using this module are meant to be a support for the teachers, helping them gain new insights on the progress of the students or by simplifying their work by automating some of the most time-consuming tasks.

As cleAR is used to create AR applications, the data collected are typically of three types: *natural text data*, for example all the data collected from chats or from answers to in-app questions; *structured data* such as all the logs collected from the applications, organized in tables where each data point represents a student interaction; and *image data*, which is data collected through interactions with the augmented content or directly from the mobile device camera. The analytics module is able to work with all these different data sources, and create models using both the supervised and unsupervised learning paradigm.

The ML algorithms are trained and stored on the server-side. cleAR allows both training of a model from scratch, or fine-tuning an existing model when new data is available. As cleAR provides a standardized API for data input, it can work with any AI framework such as PyTorch or scikit-learn, and it supports a wide range of ML algorithms such as deep neural networks or random forests. Nonetheless, in the survey, the teachers expressed the desire to understand how and why a model outputs its decisions, so we recommend developers to rely on more explainable AI models such as decision trees or linear regression models.

As the training and deployment of the models is done on the server, the client is responsible for sending the data collected to the server, and for this, cleAR provides a specific API. The client also includes tools for exploratory data analysis that allows cleaning and filtering the data in order to generate insights that can then be displayed using the visualization module described in Sect  4.4. Finally, the client also includes a set of functions for the optimization of app parameters. These functions allow, for example, to adapt the application to the hardware available or the network conditions, so that the developers do not have to take care of this beforehand. An example of optimization is described in Sect. 5.

Not every teacher who answered the survey was interested in applying automatic analysis of the data (and some of them were skeptical about the usefulness of it), but about 85% of the respondents identified the most important insights the AI-based analytics module should provide. First of all, the teachers would like to retrieve information which would be hard to come by: they are interested in finding patterns in the way the students perform so that they can plan the structure of the lessons better. For example, teachers would like to have a model that, given the results to some in-app quizzes and the time when the tests were taken, predicts the time of the day the students are more focused. This could help teachers plan their daily activities.

Our architecture is AI-agnostic, as it supports any algorithm—supervised or unsupervised—that can be implemented using standard machine learning libraries. An example of a model that can be created using the functionalities provided by cleAR is a model that predicts an average score of a student on the test on a specific subject. In this case, the AR application should collect data (which is sent in the form of xAPI statements (Clarke et al. 2020)) about how the student has been using the app. Data such as time spent on each lesson, number of interactions with the app, how quick the student was answering questions during the AR experience and so on will represent the predictor variables, while the results of the test at the end of each lesson would represent the target variables. Once the data from every student has been collected, we can then train a classification model which, when given as input the predictor variables from a new student, will predict his test results on the lessons the model has been trained on.

Another category of models that is relevant for the teacher is that of unsupervised learning algorithms, especially outlier detection and clustering models. In the first case, detection of outliers can enable teachers to flag specific content (daily activity, test results or others) as outside of the standard data distribution and then decide what to do about it. In the second case, the teacher may be interested in grouping the students into different clusters, based on the metrics she consider relevant. By tracking the structure of the clusters over time, they can keep track of how the students are progressing. Finally, AI models can also help detecting which students are “falling behind” and are having learning difficulties. Being able to identify these students at an early stage allows teachers to tackle the situation better and in a more effective way. In this case, the AI model will use metrics from both the logging data and the application usage data to train a classification model. As both the input data and the model parameters are stored on the server-side of the architecture, the models could be continuously improved using online model learning, while the data could even be combined to extract insights at classroom or at school level.

The tools developed in this module are not meant to replace the insights from the teachers and their experience based on daily interactions with the students. They are meant to be used as a support tool, helping teachers make decisions based on more data evidence and simplifying their work for more time-consuming tasks such as test grading.

### Visual reporting

The final module provides visualization and reporting functionalities. Through this module, the user can access a web interface where data can be displayed either as text or via interactive plots. This module allows teachers and students, who may not have the required expertise, to visually display the data collected and to help them draw insights from it.

**Fig. 3:**
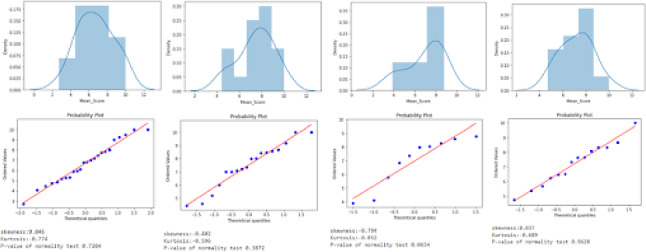
An example of data visualization created using cleAR

This module relies on existing libraries for data visualization such as D3 (Bostock et al. 2011) or Seaborn (Waskom 2021), but it simplifies the process of creating plots by providing an API for importing the output of the ML models, as well as a dashboard for generating interactive charts without code. Figure  3 shows an example of a visualization created using data collected from xAPI statements sent from an AR application to the server. The visualizations are generated on the client side. They can later be stored on the server or exported to a database, to a local storage or to the school LMS.

The visual reporting module is not meant to replace existing dataviz libraries, but rather provide teachers with a web interface that allows them to create, modify and export visualizations without requiring coding experience or directly manipulating the input data.

## Implementation and validation

We created three PoC applications based on cleAR in order to validate that the software developed using this architecture satisfies the design objectives defined in Sect. 3: *AR Cube*, *xAPI Data Analysis* and *AR Geography Quiz*.

### AR cube

**Fig. 4:**
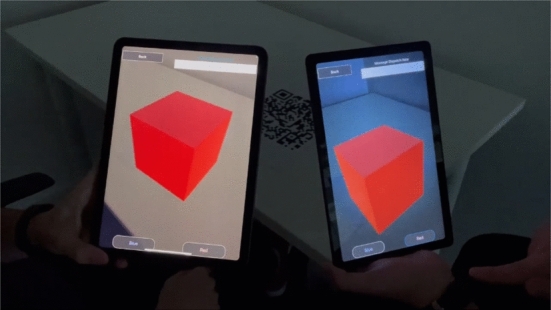
The AR Cube PoC accessed by two users sharing the same AR marker.$^4$

When starting the application, the users will join a room and all the interactions with the virtual object are broadcast to all the users connected to the same room. The user is also able to select how often data is shared between users by selecting the time interval between the messages to the server. While simple, this application demonstrates how the architecture is able to fulfill the design objectives (Fig. 4). The application is interoperable as it has been compiled for iOS, Android, Windows and Linux platforms and users are able to share their AR experience when using devices running any of these operating systems. The application supports multi-user interactions in an AR environment, as the interactions with the augmented content are the same for all users. The application has been tested for up to four users, connected through the same Wi-Fi network or on a 4 G mobile connection. For the tests, we used two tablets (a Samsung Tab A7 SM-T500 with 3Gb of RAM and a $4^{\hbox {th}}$ generation iPad Air with 4Gb of RAM) and two smartphones (a Samsung Galaxy A22 5 G and a Samsung Galaxy A90 5 G, both with 6Gb of RAM and running on Android 11). The average latency (measured as the time between when a request is sent from a user and when it is received by all users) was 205 milliseconds. There was no appreciable difference in latency between Wi-Fi and cellular network, but for messages sent from a mobile phone the latency was significantly lower than the one measured for messages sent from a tablet (mean value of 96 ms vs. 245 ms). For this PoC, the latency value was not affected by the number of clients connected or the amount of messages exchanged by the end users. For more complex applications, the resources allocated in the backend should be properly tuned in order to guarantee the desired latency.^4^

One of the parameters of the application is what we called the *dispatch time*, which is the time interval between two consecutive messages from the same user. For interactions generating many events (such as the rotations of the cube when swiping the screen), the user could generate up to 30 events per second. In order not to slow down the application (or even to saturate the network, for more data intensive applications), events are stored in a queue and then sent together once the dispatch time has elapsed. This way, it is possible to find a balance between the smoothness of the rotation and the amount of events sent. When selecting a reasonable dispatch time value (from 0.01 to 0.04 s), every user was able to experience a very smooth cube rotation. The dispatch time could also be set automatically by the application: if the user does not select a value, the app estimates the latency (by measuring the time elapsed between sending a message and receiving it back) and modifies the dispatch time accordingly until the desired latency value is achieved.

Finally, the PoC shows how easy it is to develop for cleAR. The object model properties, the application logic and the integration with the library for multi-device access required less than 400 lines of code. To enable multi-user interactions in the application, the developers only needs to register the events that affect all the users (the rotations and the color changes, in this PoC), to add a call to the function that generates a notification for these events, and to create a subscriber object which receives the notifications and modifies the app context which will then propagate the information to every user.

### xAPI data analysis

**Fig. 5:**
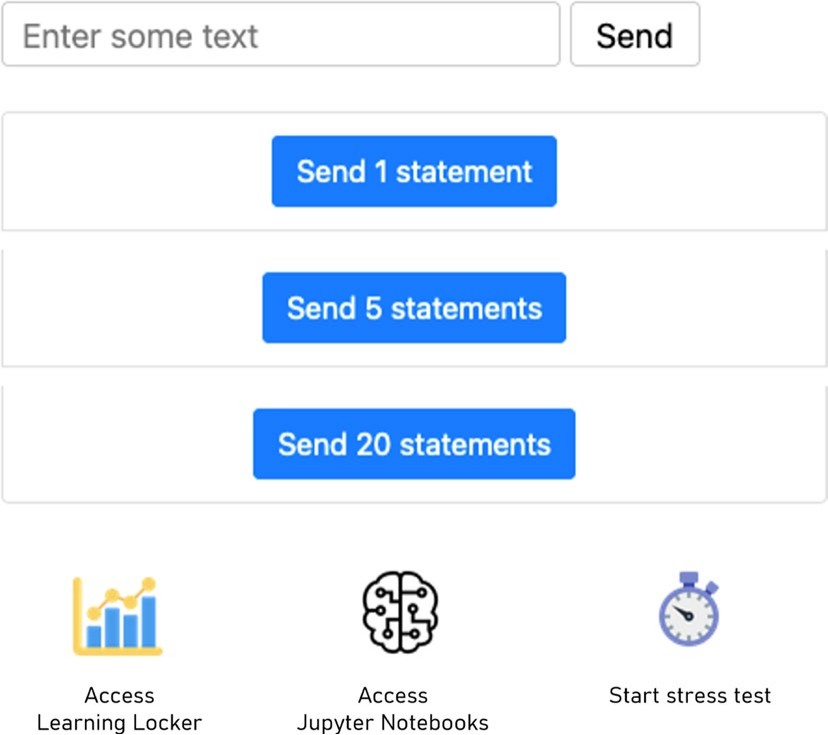
The user interface of the xAPI Data Analysis PoC. Statements are generated manually or automatically, by starting a stress test or by tracking user activity. Additional UI elements allow accessing the Learning Locker where the statements are stored as well as the notebooks used to create the AI models

The second PoC features a LRS that collects data from users accessing a web page (Fig. 5 shows the UI of the app). The application keep tracks of both active interactions (mouse clicks, text entered, etc.) as well as passive ones such as time spent on the page or date of access. The data is collected in the form of xAPI statements, which use a vocabulary specifically created for the demo application. The server-side uses Learning Locker, an LRS that supports xAPI, and all statements are stored in a MongoDB. This PoC simulates the process of data collection that could be performed in a generic AR application and was built to test the integration of cleAR with LearningLocker and scikit-learn, as well as the ability of the architecture to handle huge volumes of data without appreciable delay. Apart from the client interface for the user, another web page allows the statements to be downloaded, possibly applying different kind of filters, in a JSON format for further processing. Furthermore, a script performs weekly incremental backups of the database, copying the statements from the AWS instance where the LRS is running to a local storage.

Learning Locker provides an interface for filtering the data and for the creation of dashboards for data visualization. This way, a user can explore and visualize information without having to write a single line of code. Besides the tools offered by Learning Locker, we developed a set of functions for data cleaning, data exploration, data modeling and data visualization. These functions allow more experienced users to get more insights than the ones provided by Learning Locker and to run classification, predictive and clustering algorithms. Even though the code has to be modified and adapted for each application, using xAPI as a data format allows the creation of a standard set of library calls that favors data reuse.

To measure the ability of the deployed solution to handle the processing and storage of xAPI statements, we performed a stress test where 10 clients were generating multiple statements per seconds. A short summary of the testing conditions is available in Table 2. In total, during the test the clients sent close to 80,000 statements to the LRS, and the average delay between the sending of a statement and its availability on the LRS was 145 ms, with a maximum delay of 314 ms.

The statements generated during the stress test were also used as training data to create a simple ML classification model. The data storage and AI analytics modules of the architecture were used to fetch the data and perform the preprocessing step, which parses the data stored as json to extract the relevant input features. The independent variables used to train the model are extracted from the triplet <actor, verb, object> associated with each xAPI statement, as well as additional information such as the time delay between consecutive statements. The only statements used to train the model were the ones using “sample” as their verb, and a typical statement would be for example < *client-01*, sample, 1.04>. The object value for these statements is a number sampled from a Gaussian distribution whose mean and variance depends on the client that generated it. The statements were preprocessed to obtain a two-column input matrix—*ID* and *sample*—which could be fed to a linear classifier. Later, at test time, the model was able to successfully predict which client generated a specific statement, based on the value passed in the object of the statement triplet.^5^

**Table 2:** xAPI statements sent during the stress test of the second proof-of-concept app

Test	Statements/batch	Wait time	Statements
1	15	10 s	1653
2	30	5 s	6614
3	30	2 s	16,534
4	50	1 s	55,116
Total			79917

**Fig. 6:**
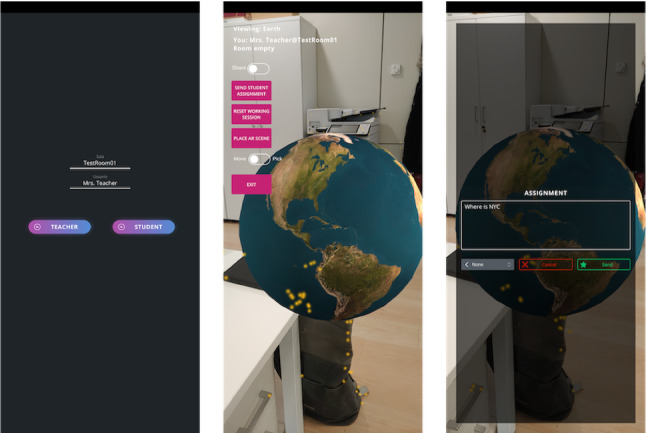
The AR Geography Quiz application. From left to right: the login screen, the augmented content, the “send question” view.$^5$

**Table 3:** Summary of design objectives fulfilled by each proof-of-concept application

Proof-of-concept	Design obj.				
	Interoperability	Multi-user	AI support	Data storage	Easy to develop
AR cube	$\checkmark$	$\checkmark$	$\checkmark$		$\checkmark$
xAPI data analysis		$\checkmark$	$\checkmark$	$\checkmark$	$\checkmark$
AR geography quiz	$\checkmark$	$\checkmark$		$\checkmark$	

### AR geography quiz

The last PoC implemented is a more complex AR application that simulates an interactive geography quiz which, for example, could be used in a classroom to evaluate the knowledge of the students regarding subjects they recently studied (see Fig. 6 for an example of what the teacher interface looks like). In this example, we use AR Foundation to create an AR scene in unity where the user sees a 3D model of the Earth and she can change what she sees by swiping the finger on the display or by physically moving around the 3D element. In the application, one teacher and one or more students connect to the same virtual room to participate in the quiz (the video linked in footnote 5 only shows interactions between the teacher and one student, for the sake of simplicity, but the PoC also supports one-to-many interactions). The application works in two modalities. In the first one, each user can freely explore the augmented content, either by rotating the globe or by actually moving around it, and there is no shared experience between users. In the second modality, one user can force every other user to watch the globe from their perspective, and it is in this modality that the users are sharing their interactions. For example, the teacher has the ability to force every user to see the 3D Earth from his point of view (PoV), forcing the AR camera position to be the same for all users, and to send questions such as “Where is Canada?” to a specific user. When that happens, the student who received the question will then share their camera PoV (effectively controlling what other users are seeing on their device) and he can answer the question by placing a marker on the globe. Once that is done the teacher will re-gain control of the application and mark whether the student answered correctly. A multi-user AR application for education can make the learning experience more engaging and promote collaboration between users by enabling interactions with the environment and also with other students.

This PoC uses the same library for multi-user interactions developed for the first PoC, and it has been compiled for both desktop and mobile (Android and iOS) platforms. A server is used to store and forward all the events and messages passed between the clients, and an online database is used to store the questions, answers and the progress of each student.

The PoCs developed demonstrate how cleAR fulfills the design objectives identified in both the literature and the conducted survey. Table 3 summarizes which design objectives are satisfied by each PoC. AR Cube is clearly interoperable, as it has been tested on several operating systems. It also shows that multi-user capabilities can be easily integrated into any application. This PoC also implements an AI-based algorithm that automatically sets the value of the *dispatch time* based on the current network conditions. The xAPI Data Analysis PoC is a web-based application which allows multiple users to interact and stores the interaction statements in a learning record repository. Enabling storage of xAPI statements is straightforward, and AI support is guaranteed by the implementation of classification models which use the data gathered from the recorded statements as input features. The last PoC, AR Geography Quiz, is more complex than the first two PoCs. It has been developed for both web and mobile platforms and is inherently multi-user. It also demonstrates that cleAR enables data storage in the developed applications as all the interactions, as well as the questions and answers, are stored.

## Conclusions and future work

In this paper, we described cleAR, an architecture enabling the creation of interactive and collaborative AR applications for education. To define the design objectives of cleAR, we performed a survey of the existing literature on the subject and gathered the architecture requirements from a survey completed by primary and secondary school teachers. cleAR is composed of four different modules, responsible for enabling multi-user interactions, data storage, data analytics and visualization. We created three demo applications to demonstrate that the architecture complies with the design objectives. We believe that cleAR will help developers in the creation of AR applications that could be easily included in existing school curricula. This in turn will provide the teachers with a suite of tools that enables them to keep records of student activity, add smart analytics and automatically create reports about student progress and retention.

The architecture presented is interoperable and can work with most of the software suites currently in use to produce AR applications. Since it is modular, developers can choose which parts of the architecture should be integrated into existing applications. As the majority of existing AR apps are client only, the most critical aspect for integration with existing software is the provision of a server which provides all the desired functionalities. Based on our experience, we would recommend at first to integrate the data storage and visualization modules, and only later add multi-user and AI functionalities.

Future research directions include the development of a complete implementation of cleAR and the development of an educational AR application which fully supports all the functionalities of cleAR. We plan to complete the development of an app and validate it by conducting user tests in several schools, and to assess its usability and the user experience by using standardized questionnaires (Brooke 1996). Furthermore, we are investigating how to extend cleAR to provide authoring functionalities. An authoring tool will streamline and simplify the creation of AR applications and allow teachers to create their own content without relying on external developers, thus encouraging the inclusion of AR apps in school programs even more.

## Data Availability

The datasets generated during and/or analyzed during the current study are available from the corresponding author on reasonable request.

## Footnotes

1 https://github.com/Stocastico/ARchitecture_paper.

2 https://anon.to/oBIuF6.

3 https://anon.to/3vzfK4.

4 A video demo is available at https://anon.to/pwRr4W.

5 A video demo is available at https://anon.to/4Ulhto.
